# Association of Ideas

**Published:** 1868-09

**Authors:** 


### Hall's Journal of Health, September, 1868.

### ASSOCIATION OF IDEAS.

"Once ago," as our then little Robbie used to say; we were emptying our waste box and observed a sheet of paper crumpled up and covered with pencil writing; on examination, it was found to be the first rough draft of a composition written by our youngest daughter; we printed it verbatim et literatim in the July Journal of Health, without note or comment, intending in the next number to make a practical use of it, in reference to some of the laws of mind and matter. The Editor of the EXAMINER AND CHRONICLE of New York City, who makes one of the very best and ablest Religious newspapers in the United States, in scissoring for his issue for July 16th, 1868, found nothing so worthy of use in all our July number as the article THE OLD SCHOOL HOUSE "by grandmother Howell" who wrote it at the age of "Three score years and Ten," as the story goes. Now, here is a Reverend D. D. who stands a head and shoulders above most of his craft in ability and worth, deciding that an article written by a girl of thirteen, of the Twelfth street public school, the first rough draft of it mind, written on her knee, in our office, without the slighest aid or suggestion from any one, as to subject, expression, spelling or anything else, because most strictly forbidden by the rules of the school, so as to throw the children on their own resources, as the best means of self developement; that Dr. Bright, we repeat, should decide that the composition of a school girl of thirteen should be more worthy of being copied into his paper, than anything which we, at the age of Methuselah, more or less, had written, is a matter worthy of note.

When it is taken into account that this child had never seen an "old red school house" in her life, and had coined this whole story out of her own brain, as if she were writing it under the influence of supposed reminiscences, from the standpoint of seventy years, certainly impressed us deeply, as to the wonderful nature of the human mind, which can not only bring up the feelings and impressions of the remembered past, but can conjecture what will most probably be its feelings and impressions half a century to come. It is so few years ago, when this same child was walking down 5th Ave. every morning to her school, with her soft little warm hand in her father's, her dark curls hanging over her shoulders and her trusting loving face turned up to her father's every few steps, to ask some childish question, or to listen to some sage remark, every syllable taken as gospel! She had grown up beyond her father's height, causing her to be addressed as "My Darling Bean Pole," and we could not separate the ideas in our own mind; they would come upon us in the shape, that it was the little chatty child who wrote the composition; as the mother, who has early lost her first born, continues to think of it in all after life, as she last saw it; and feels too, that when she gets to heaven, she will recognize it there, by the same face it had in its infancy, when it was not dead, but transplanted! The young man separates from his classmates at college; for thirty or fifty years he does not meet with some of them again; but whenever he thinks of them after that long interval, the face and form and features of "commencement day" stand out before his mind's eye, like a vivid reality. And many times in passing the crowded streets, we see a face so strikingly like that of our classmate, as it was at parting, that we are almost tempted to call him by name, although half a century has intervened; so difficult is it to make up our mind to the fact of CHANGE!

Thus did our thoughts run on, by the influence of the "Association of Ideas," when we read our little daughter's article about "The Old School House," and we thought we would make use of it in the very next number of our Journal. But who can tell what a day may bring forth! Months have passed away, and they have brought other changes upon us, which shall materially affect the whole aspect of future life, and even give color to our destiny beyond. Dies have been cast which are irrevocable for all time to come; breaking up our whole intended career; opening up new vistas, which but a few months ago we did not dream could come within the range of human possibility ; and yet, when the whirlwind of sweeping changes has passed along, we quietly, by the aid of that inexplicable principle of "Association of Ideas," take up "the thread of the discourse," as if nothing had ever happened beyond the usual humdrum routine of life. But the restless mind did not stop there. The little child was connected with the woman's composition ; the infant's face in the mother's mind, with its identity beyond the Jordan of death ; the classmate's features unchanged in the memory of the old man of four score. But another thought was linked on to all the above, beautiful and blessed to contemplate, the mother's instinct, the classmate's instinct, are against "CHANGE." Can any instinct be falsely planted in us ? Can we have false instincts ? We feel as if these came from the hand of Divinity itself, and Divinity is always beneficent and always true. Then it would seem to follow, without any violence in the deduction, without any link in the chain being broken, that the face, the personality, which we have at death, will be immortal ; will be eternal ; will never change, except, it may be, in the way of refinement, or a species of exaggeration, which the artist throws upon his painting, and which without destroying the resemblance, actually makes the picture more like the original. In plainer phrase, we shall not grow old in heaven ; but will be always as young in feature and form, as when we left the world ; and not only always young, but always WELL, always happy and always good.

In the item of immortal youth there may indeed be no change, there will be changes even in Heaven, but they will be from glory to glory. When "the earthly house of this tabernacle is dissolved," when this body faints and fails and turns to dust, under the destructive influence of age and disease, we shall arise to a "newness of life," shall be endowed with a renovated form, "incorruptible, undefiled and that fadeth not away," a form that shall be always young and always WELL. And with the aid of eternal health and eternal youth, in that genial clime, where "death itself is dead," and with the aid of the thought that as death leaves us so will the judgment find us, at least in one sense, the reader can easily see through the connection, which the association of ideas forms, that those whom death finds engaged in the culture and practice of "love to God and love to man," "of joy, peace, long suffering, gentleness, goodness, faith, meekness, temperance," will enter upon an existence where, with immortal youth and health perennial, they shall grow, shall expand more and more into the likeness of Him who is the embodiment of all perfection, human and divine. And in view of considerations like these, who shall not say, that they are wise whose highest, chiefest aim and business in life, is to commence here and persist until the last summons comes, to cherish and cultivate and practice all those traits of heart and character which make a man akin to angels; in other words, who shall not say, that it is worth the effort of a life time. to be able to die well, "whom his lord when he cometh shall find so doing." All know that it is age and disease which arrest the progress and improvement of all men; but it is the fewest of the few who practically feel and believe that it is possible in this life to lay the foundation of an eternal existence of health and youth, where the head and the heart, where the mind and the affections shall enter upon a state of progression which neither disease nor age can ever interrupt and which ends not short of the infinite. The ancient philosopher asked his pitiless captor in vain to spare his life for one brief moment, that he might complete his demonstration. "Look and Live" forever, are the generous terms held out to mortal man. Euclid asked in vain for the luxury of one more moment of progress; most men in these ages, neglect the offer and the terms, of an infinite duration of progression. Reader! art thou one of these?

